# Success and failure of dead-time models as applied to hybrid pixel detectors in high-flux applications

**DOI:** 10.1107/S0909049513000411

**Published:** 2013-01-30

**Authors:** B. A. Sobott, Ch. Broennimann, B. Schmitt, P. Trueb, M. Schneebeli, V. Lee, D. J. Peake, S. Elbracht-Leong, A. Schubert, N. Kirby, M. J. Boland, C. T. Chantler, Z. Barnea, R. P. Rassool

**Affiliations:** a School of Physics, The University of Melbourne, Melbourne, Victoria 3010, Australia; b DECTRIS Ltd, 5400 Baden, Switzerland; c Paul Scherrer Institut (PSI), CH-5232 Villigen, Switzerland; d Australian Synchrotron, Clayton, Australia

**Keywords:** hybrid pixel detector, dead-time, single-photon counting, synchrotron fill pattern

## Abstract

The performance of a single-photon-counting hybrid pixel detector has been investigated at the Australian Synchrotron. Results are compared with the body of accepted analytical models previously validated with other detectors. Detector functionals are valuable for empirical calibration. It is shown that the matching of the detector dead-time with the temporal synchrotron source structure leads to substantial improvements in count rate and linearity of response. Standard implementations are linear up to ∼0.36 MHz pixel^−1^; the optimized linearity in this configuration has an extended range up to ∼0.71 MHz pixel^−1^; these are further correctable with a transfer function to ∼1.77 MHz pixel^−1^. This new approach has wide application both in high-accuracy fundamental experiments and in standard crystallographic X-ray fluorescence and other X-ray measurements. The explicit use of data variance (rather than *N* ^1/2^ noise) and direct measures of goodness-of-fit (χ_*r*_^2^) are introduced, raising issues not encountered in previous literature for any detector, and suggesting that these inadequacies of models may apply to most detector types. Specifically, parametrization of models with non-physical values can lead to remarkable agreement for a range of count-rate, pulse-frequency and temporal structure. However, especially when the dead-time is near resonant with the temporal structure, limitations of these classical models become apparent. Further, a lack of agreement at extreme count rates was evident.

## Abstract 2

Detector response functionals are found to have useful but also limited application to synchrotron studies where bunched fills are becoming common. By matching the detector response function to the source temporal structure, substantial improvements in efficiency, count rate and linearity are possible.

## 1. Introduction   

Fluorescence X-ray absorption fine structure (XAFS), small-angle X-ray scattering (SAXS) and protein crystallography are important applications of synchrotron radiation that require the position and relative intensity of X-rays to be determined to high accuracy. Widespread use of area detectors for high-throughput crystallography, where the weakest reflection, the strongest reflection and the curve of the diffraction spot profile cover many orders of magnitude of flux and brightness, leads to this being a critical consideration. Further, the temporal structure of recorded spots introduces yet another time dependence to the source. A few attempts on laboratory diffractometers have investigated the absolute calibration and hence linearity of diffracted intensities relative to the straight-through beam (Harada *et al.*, 1970 ▶). This necessitates the use of detectors with high radiation tolerance, high dynamic range, low noise performance and a small point spread function. Single-photon-counting pixel array detectors (PADs) such as PILATUS have demonstrated an ability to meet these criteria (Broennimann *et al.*, 2006*a* ▶; Sobott *et al.*, 2009 ▶).

Many other synchrotron applications benefit from these advanced characteristics. Moreover, these advantages serve well in high-flux operation, including measurements of direct-beam or attenuated beam geometries, but also in medium or low-flux operation, including scattering and fluorescence detection from disordered or dilute systems. A range of critical experiments including tests of QED (Pohl *et al.*, 2011 ▶; Gillaspy *et al.*, 2010 ▶; Chantler *et al.*, 2009*a* ▶) also depend upon such characteristics of the detector chain. Too often the best measurements are limited by either statistics (detector efficiency and count-rate) or by systematic errors including non-linearities (Chantler & Kimpton, 2009 ▶). Hence even modest advances in these areas can lead to dramatic new science. In fact, in several of these fields, an increase in final accuracy by even a factor of two provides valuable insight into vacuum fluctuations and higher-order Feynman diagrams (Hudson *et al.*, 2007 ▶).

Inherent in all photon-counting detectors is a time period after a recorded event where the detector is rendered insensitive to further events. This dead-time is due to the finite time required to process each pulse (Johnson *et al.*, 1966 ▶; Reed, 1972 ▶; Sharma & Walker, 1992 ▶) and if the detector response profile is known then a simple correction for the dead-time can be applied. Numerous analytical models assume a continuous X-ray flux; however, many synchrotron fill patterns employed around the world are highly structured. In such cases the correction for dead-time, and the avoidance or reduction thereof, becomes both more interesting and complex. Analytical solutions taking into account the temporal structure of the source have been previously presented (Cousins, 1994 ▶; Kishimoto, 1997 ▶).

We describe below the dependence of maximum detector count rate and linearity on the temporal structure of a storage-ring fill pattern and detector dead-time. Results are compared with hitherto accepted models and potential causes of deviations are discussed.

## 2. Analytical models   

Two cases of source flux have been discussed and modelled analytically in the literature, that of uniform fill and of bunched fill. While other cases of arbitrary complexity can be modelled using, for example, Monte Carlo methods, in this investigation we explored the exemplars and fundamental ideas *via* suitable analytic formulations.

### 2.1. Uniform synchrotron fill   

In the case of a uniform fill each bunch contains an almost identical number of electrons and the photon arrival rate can be considered uniform (*i.e.* Poissonian). A uniform synchrotron fill is an idealization, both experimentally and theoretically, and perhaps might best be modelled with a rotating-anode source.

For a non-paralyzable detector, a signal above a simple discriminator leads to a simple response of the counting system to an incident X-ray rate driving the system, 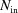,
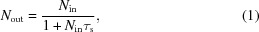
where 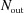 is the observed rate and 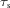 is the dead-time including intrinsic detector and electronic components (Knoll, 1989 ▶). Consequently, when the dead-time is constant over all events and events are random in time (Johnson *et al.*, 1966 ▶; Reed, 1972 ▶; Sharma & Walker, 1992 ▶), a relatively simple correction factor can be applied to correct for non-linearity of response.

For a paralyzable detector, each photon resets the time during which the detector is insensitive to photons and the observed rate is described by (Walko *et al.*, 2011 ▶)
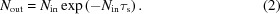
Notice that this model results in paralysis, that is, an increasing incident count rate will result in a lower observed count rate. The dead-time is an *effective* dead-time, as the signal loss may not correspond directly to the dead-time setting on the amplifier but rather is a function of the entire signal processing chain (namely intrinsic and electronic contributions to dead-time).

A third situation may be defined where the paralyzable detector pulse is rejected if the pulses are distorted by pile-up, for example if pulse height analysis (PHA) is performed (Bateman, 2000 ▶). The observed count rate can then be described by 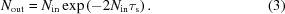
This is similar to (2) but the onset of paralysis is ‘twice as fast’ since the distorted peak does not count as one count but as zero (it is rejected). At high count rates the ability of a discriminator-based system to recover from pile-up and return below threshold is decreased. The losses due to dead-time have a much faster onset because the pulse length must remain undistorted.

### 2.2. Bunched synchrotron fill   

The introduction of single bunches into the beam structure allows the response of the detection system to short bursts of photons arriving at regular intervals to be studied (Honkimäki & Suortti, 2007 ▶). If the interval between bunches (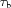) is greater than the intrinsic dead-time of the detector then the observed count rate is dominated by the bunch spacing. In this case the expected counts from a discriminator-based system, a paralyzable detector or a pile-up rejection system can be described, respectively, by (Bateman, 2000 ▶)
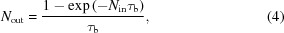


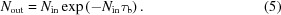
This is sometimes called the ‘isolated model’, noting that the shaping dead-time of the detector is irrelevant to the response function. Similarly, if the bunch spacing is reduced to less than the intrinsic dead-time of the detector, *i.e.* 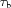 < 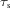, the expected counts from a discriminator-based system, a paralyzable detector and a pile-up rejection system can be described, respectively, by
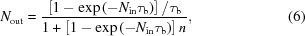


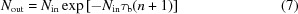
and
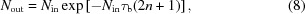
where *n* is an integer defined by *n* = 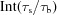 and describes the discrete nature of the source. For the case where *n* = 0, or 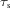 < 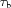, (6) reduces to (4) and both (7) and (8) reduce to (5).

In addition to investigating the expected benefits to detector linearity from the introduction of a bunched fill, we investigate accepted analytical models as the relation between detector dead-time and bunch spacing approaches resonance.

## 3. Experiment   

### 3.1. System description   

The PILATUS detector system has been described in detail (Broennimann *et al.*, 2006*a* ▶). Briefly, each PILATUS module comprises 94965 square pixels of side length 172 µm, creating a continuous detector area of 83.78 mm × 33.56 mm. Each pixel comprises the necessary electronics to process and record individual events. Charge liberated in the sensor by incident radiation is transferred to the readout *via* a microscopic bump-bond (Broennimann *et al.*, 2006*b* ▶). The signal is subsequently amplified and shaped before discrimination against a pre-determined threshold. If the incident radiation deposits sufficient charge, a local counter is incremented, leading to a complete digital storage of the number of detected events at the pixel level. External bias voltages allow the dead-time of the preamplifier and shaper to be optimized with respect to energy resolution or speed, dictated by the constraints of the experiment. All data presented in this report were acquired with PILATUS; however, results are applicable to any lower-level discriminator-based detector system.

### 3.2. Australian Synchrotron   

The Australian Synchrotron is a third-generation light source (Boldeman & Einfeld, 2004 ▶) possessing the key characteristics outlined in Table 1 ▶.

**Figure 1:**
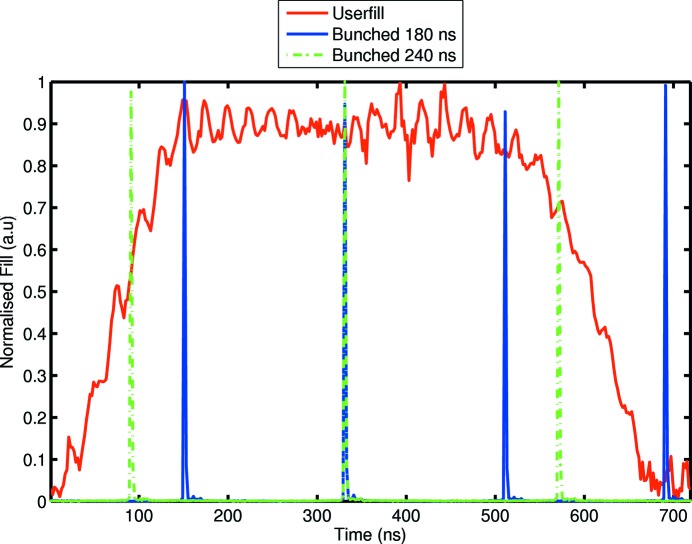
The standard user fill, 180 ns bunched and 240 ns bunched patterns as measured with the fill pattern monitor.

Investigations were restricted to fill patterns comprising integer divisions of the revolution time, whilst providing a temporal interval comparable with relevant detector dead-times. Consequently, data were acquired for single-bunch injections with separations of 180 ns and 240 ns. Reference data were also acquired with the standard user fill pattern shown in Fig. 1 ▶.

### 3.3. Measurement   

Measurements were undertaken at the Australian Synchrotron Small-Angle X-ray Scattering/Wide-Angle Scattering beamline, utilizing 16 keV radiation. An EPICS (Experimental Physics and Industrial Control System) script was implemented to translate two sets of aluminium attenuators across the field of view of PILATUS. The first set comprised four and the second set 14 attenuators of increasing thickness, resulting in 56 applicable attenuation factors and data points for each shaping time.

Reference images were obtained in the linear region [less than 10 kHz per pixel (Kraft *et al.*, 2009 ▶)] of the detector for each attenuator thereby allowing determination of each attenuation factor. The attenuation factor was subsequently used to determine the true incident photon rate from the detected incident photon rate. The accuracy of this low-flux determination is approximately 1–2%, quite adequate for the investigation presented herein, as evident from the data (see §4.1).

Higher-order undulator harmonics have the potential to deposit substantial charge in the sensor and can therefore severely affect detector counting capabilities (Barnea *et al.*, 2011 ▶). A multiple-foil method has been previously applied to quantify the harmonic fraction at <0.01% (Tran *et al.*, 2003 ▶), which is further reduced by the use of a Si(111) double-crystal monochromator (DCM). The low harmonic content of the 3 GeV ring, 0.1%, combined with the DCM reflections at approximately double the critical energy, enabled the harmonic content to be calculated as 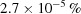. PHA data from an individual pixel were used to confirm the absence of any substantial harmonic contamination.

In order to avoid counter overflow whilst maintaining adequate statistics, ten sets of 100 ms exposures of the de­focused beam were acquired with each attenuator combination. This process was repeated for three shaping times with a standard user fill pattern and for seven shaping times with bunched fill patterns. All data were acquired with a 50% incident energy threshold on PILATUS (Broennimann *et al.*, 2006*b* ▶) and the response of a single representative pixel is presented in §4. During all acquisitions the electron distribution within the storage ring was observed *via* a fill pattern monitor (FPM) (Peake *et al.*, 2008 ▶). Implemented on the optical diagnostic beamline (Boland *et al.*, 2006 ▶), the FPM utilizes a metal–semiconductor–metal (MSM) diode to measure incident optical flux and hence infer the stored electron distribution.

## 4. Analysis   

### 4.1. Model validation for standard or uniform fill pattern   

In order to study the relationship between detector response and beam structure, three fill patterns were investigated. Validation was initially performed with the standard user fill, which comprises 600 ns of trapezoidal fill isolated by a 120 ns gap. Data were acquired at shaping times ranging from 125 ns to 383 ns for single buckets separated by 180 ns and 240 ns. The three fill patterns as measured with the FPM are shown in Fig. 1 ▶ and the corresponding temporal parameters are presented in Table 2 ▶. Summarized in Table 3 ▶ are the shaping times used in the measurements. The effective shaping time refers to the effective pulse duration and is derived from previous parameterizations (Kraft *et al.*, 2009 ▶).

**Figure 2:**
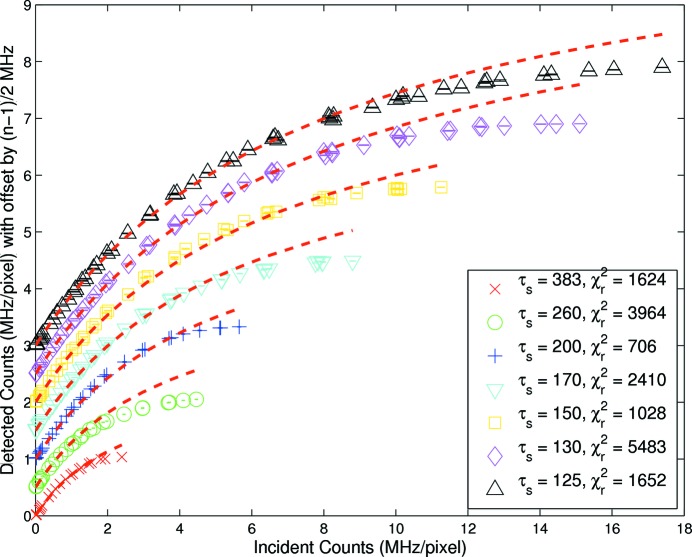
Measured rate response at each shaping time for 180 ns bunched fill. The corresponding fits are based on a 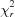 minimization of equation (1), *i.e.* uniform fill model without pulse rejection with fixed coefficient 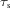. Subsequent offset on the *y*-axis for the series of nominal shaping times 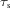 allowed the model inadequacy to be clearly seen.

**Figure 3:**
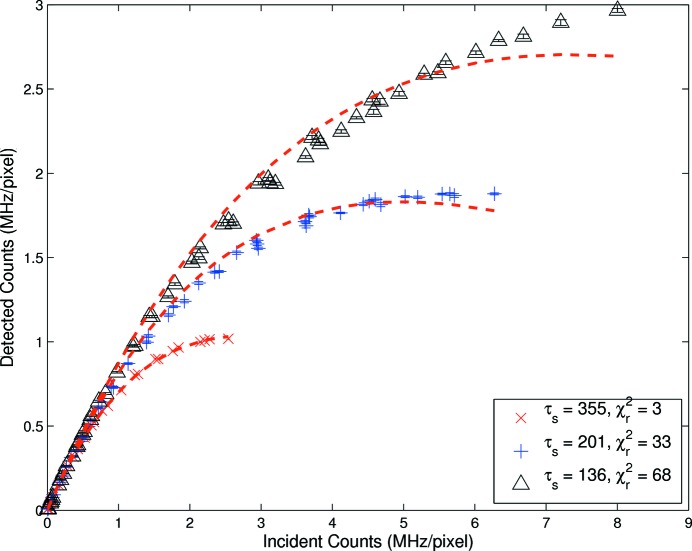
Measured rate response obtained from the standard user fill paralyzable detector with no pulse rejection [equation (2)] with shaping time as a free parameter. The largest shaping time 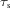 was modelled well while the smallest shaping times were clearly not modelled by the expected non-bunched formula.

In probing the models in §2, the simplest model was considered first. Results are illustrated for the 180 ns bunched fill in Fig. 2 ▶ and fits are based on equation (1), *i.e.* a uniform fill in the absence of pile-up rejection. The input uncertainty was dominated by temporal variance due to beam drift and flux variation and was significantly larger than 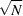 noise. Therefore the variance of repeated measurements was used to establish a reasonable and robust input weighting for analysis and to allow the determination of significance in relation to agreement or disagreement with the models previously discussed. Very poor agreement was evident and the corresponding 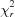 values are large. The application of equation (1) to standard user fill data, Fig. 3 ▶, also resulted in an extremely poor fit, with reduced 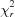 values for effective shaping times of 125 ns, 200 ns and 384 ns of 68, 33 and 3, respectively. Even in the region where this model should be appropriate, it was clearly and strongly at odds with the data. As far as we are aware, this was the first time that modelling of advanced detector responses and linearity had included explicit variance measures and evaluated goodness of fit using appropriate 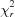 methods. This was crucial as visual inspection could interpret a good fit for lower flux rates even when the model was clearly invalid.

The uniform fill model should have been a good qualitative match for the standard user fill, but indeed the model was non-paralyzable and we therefore expected the paralyzable model to match the data. Application of equation (3), *i.e.* uniform fill with pulse rejection, failed to improve the fits and indicated that this model function was inappropriate. Incidentally, the better of these three uniform fill models was clearly equation (2), *i.e.* the paralyzable detector without pulse-pileup rejection, and this most nearly approximated the detector type and electronic operation.

To investigate the validity of the shaping time determinations, 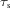 was allowed to vary for a fixed bunched spacing. We emphasize that none of these models have more than two parameters, which may be adjusted freely to provide an empirical fit; or constrained such as for a fixed bunch spacing on the assumption that the measurement was at least reflective of an effective bunch spacing within its uncertainty.

For the standard fill pattern, most appropriately represented by a uniform fill model, equation (2), the paralyzable detector without pulse-overlap rejection, the shaping times shifted from 384 ns, 200 ns and 125 ns (predicted/measured) to 355 ns, 201 ns and 136 ns (fitted). Each of these parameters was within one standard deviation uncertainty of the predicted value, and therefore justify both the choice of model and its implementation. However, while 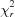 for the longest shaping time was 3, which represented a good fit, the shorter shaping times clearly did not follow this model. Something was missing, either in time structure, detector electronic processing, experimental measurement or uncertainty evaluation, or in some more fundamental understanding of the detector response function at high counting rates. The deviations were systematic and not random, suggesting a causal nature of the discrepancy. It was possible to gain a better empirical fit using a bunched model in some cases, while having two parameters free. However, the use of such a bunched model was unphysical both in model identification and in the parameter values fitted, though it can be quite useful for an empirical understanding of the functional shape at high counting rates.

### 4.2. Model validation for 240 ns and 180 ns bunched fills: optimized models only   

**Figure 4:**
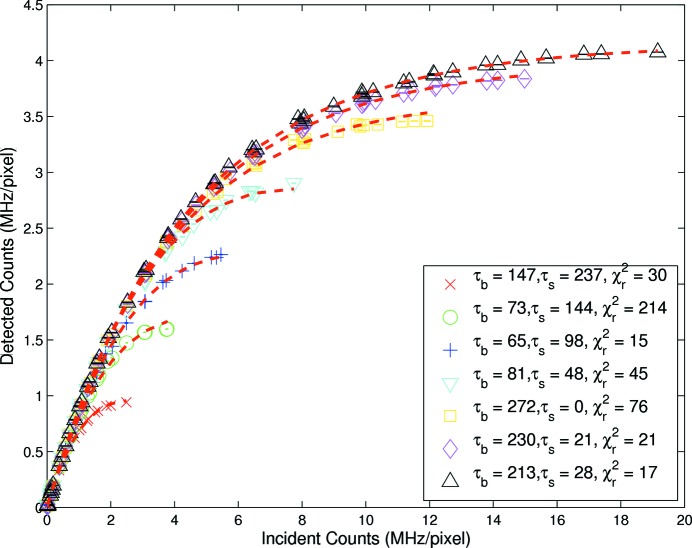
Measured rate response obtained from 240 ns bunched fill with both bunched spacing and shaping time as free parameters and listed in the key with the corresponding 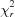. The rows are ordered in decreasing shaping time where the first four were best modelled by equation (7) and the last three were best modelled by equation (6).

Similarly, for the bunched 240 ns data, with three fills, fitting was undertaken with both shaping time and bunch spacing as free parameters. This resulted in a substantial improvement in the goodness of fit. Fig. 4 ▶ represents the best fits for each shaping time, with the bunch spacing and the shaping time as free parameters. The largest four shaping times were best modelled by equation (7) while the shortest three were best modelled by equation (6). This had an unclear physical basis; ideally all should have been best modelled by equation (7). Furthermore, the parameter values obtained were generally unphysical. It followed that this allowed empirical modelling of specific experimental data but the predictive value at this juncture was quite limited. The qualitative understanding of the functional form of the experimental data was nonetheless significantly improved.


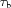
 and 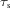 could certainly be correlated in some of the models, yielding a flat 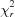 valley and therefore a great difficulty in determining the true minimum. This did not remove the difficulty of the fitted parametrization. A second important point is that the beam optics could in turn shape the bunching further beyond the measured values; while we have no evidence for this effect, it would be reflected in an empirical bunching parameter which was somewhat changed from the measured one, and not as dramatically as was observed.

Some care must be taken in interpreting the results. Arguably the best of these fits was not a good fit, as shown by the 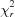 values. The 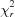 valley was sometimes very shallow; for example, for the longest shaping time (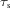 = 384 s), 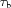 = 240 ns, a value of 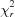 = 30 was obtained from equation (7), with parameters 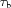 = 147 ns, 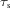 = 237 ns, but the same model with fixed 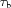 = 240 ns and 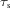 = 144 ns yielded the same 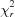, as indeed did a model using equation (2) with the single (free) parameter 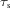 = 384 ns. In some cases the model form contained parameters which were certainly not independent.

**Figure 5:**
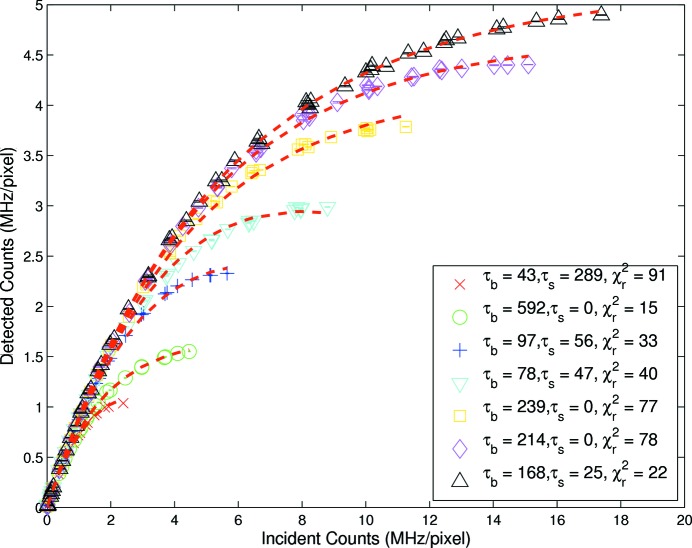
Measured rate response obtained from 180 ns bunched fill with both bunched spacing and shaping time as free parameters. Comparison with Fig. 2 ▶ indicates a substantial improvement in goodness of fit. The model of equation (7) was preferred for shaping times 1, 3 and 4 while the model of equation (6) was preferred for shaping times 2, 5, 6 and 7.

**Figure 6:**
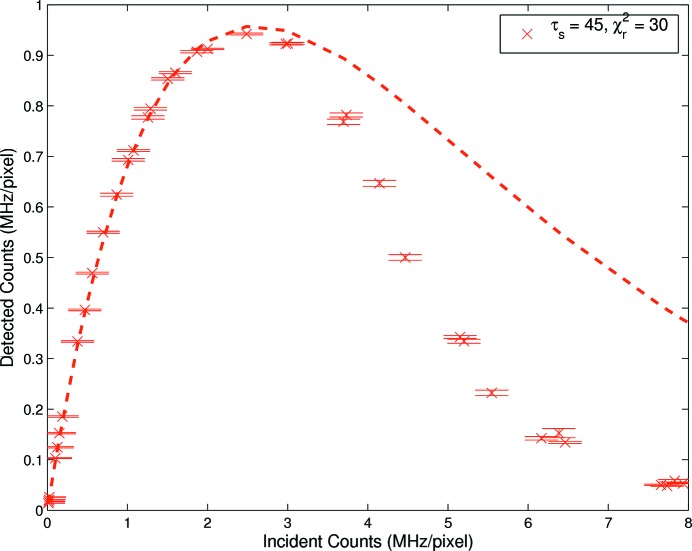
Detector rate response post turnover. A rate-dependent divergence between measured and expected counts is clearly evident.

Investigating the 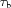 = 180 ns data revealed a similar inconsistency (Fig. 5 ▶). No single model fitted the data and some conditions were not reasonably fitted by any model, even with all parameters free. Visual inspection alone may look reasonable but all options fail if the reduced 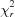 is used as the metric for goodness of fit. Therefore a new model with a more grounded physical basis is required. The bunched fill models, independent of whether the parameters were fixed or free, adequately described bunched data but the choice of models remains inconclusive. The detector was in fact paralyzable (see Fig. 6 ▶) so that over longer flux ranges the discrepancies were clear.

As a cautionary note, one should consider the nature of the *n* = 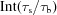 factor in Figs. 4 ▶ and 5 ▶. In fitting, the least-squares approach naturally expects continuous variables, so we have modelled the functionals of equations (6) and (7) with *n* = 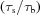. The plotted (optimal) fits therefore would be largely summarized or approximated by *n* = 1 for the three-bunch settings, 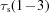, and *n* = 0 for 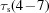. As the shaping time gets shorter, these models should correctly approach the *n* = 0 limit, and the lack of direct physical parameterization of 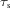 is not a proper criticism of these models. Ideally, we might anticipate a change-over of *n* around 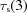 for the three-bunch data and around 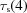 for the four-bunch data. While this was not properly observed, this aspect of the bunched models was qualitatively substantiated.

### 4.3. Model validation post turnover   

For a uniform fill pattern the maximum count rate occurs at 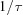, as indicated by equation (1). Increasing the incident flux above this value increases the likelihood of pulse pile-up and reduces the ability of the system to return below threshold. Surpassing the maximum count rate results in a non-monotonic relationship between incident and measured counts, thus introducing ambiguity with respect to the true incident rate. It is therefore important that detector operation is performed below the maximum count rate. However, for complete model comparison, data were acquired well past the turnover point. As seen in Fig. 6 ▶, a rate-dependent divergence between the measured and expected counts was clearly evident. There is evidence to suggest that the simple models enumerated in this study, despite being the dominant models of the literature to date, were inadequate to describe fully the operation of these detectors at very high flux.

### 4.4. Linearity   

The complex regions presented correspond to very high flux rates, and indeed empirical fits were found in all cases. However, to examine departure from linearity, a reduced region of interest was defined for Figs. 3 ▶, 4 ▶ and 5 ▶.

**Figure 7:**
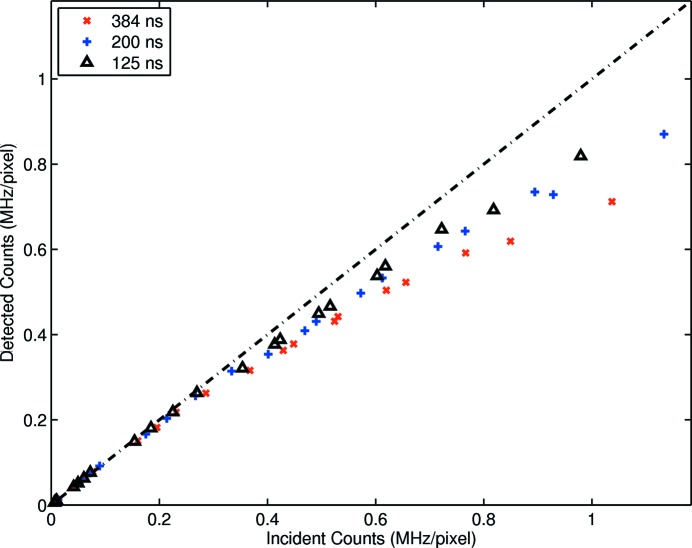
Enlargement of Fig. 3 ▶ to reveal the large linear range of the detector. Linearity to within approximately 2% is evident at short shaping times up to approximately 0.36 MHz pixel^−1^. This becomes significantly non-linear at very high fluxes. However, the introduction of bunched fill substantially improves the linearity over this higher flux range.

Corresponding results are shown in Figs. 7 ▶, 8 ▶ and 9 ▶. Results acquired with the standard user fill pattern indicate that linearity was maintained at the shortest dead-time to approximately 0.36 MHz pixel^−1^. This value was improved to approximately 0.59 MHz pixel^−1^ by introducing a 240 ns bunch time gap and to 0.71 MHz pixel^−1^ by introducing a 180 ns bunch time gap. Improvement was evident across the majority of shaping times, the exception being a shaping time of approximately 260 ns.

Despite the fill pattern producing many photons per bunch, the non-continuous structure of the fill pattern allowed detector efficiency to exceed that indicated by (1). Further, linearity, dead-time and maximum count rate were all improved by a bunched fill pattern. These results demonstrate that the implementation of rate-correction factors to maintain data accuracy outside the linear region of a detector is contingent on an *apriori* knowledge of the fill pattern. Particularly for time-structured fill patterns, any modification to detector dead-time must be coupled to an appropriate applied correction factor.

## 5. Conclusions and outlook   

The rate response of the detector has been compared with expected values from a wide range of accepted models, *i.e.* the dominant models reported across the literature of electronic detector response functions. Proper 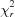 fitting has been introduced and quoted for the first time, and model agreement is specifically characterized by this measure. This has proven that 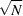 or counting noise was not the dominant cause of variance and hence input experimental uncertainties must be evaluated carefully in all such experiments and investigations.

The functional linearity of the detector chain is excellent, but is critically dependent upon dead-time. The linearity and the maximal count rate measurable with a detector chain is similarly critically dependent upon the matching of dead-time (shaping time) to the storage-ring fill pattern. We have presented all traditional models for dead-time response, and found that empirical fits across wide ranges of flux and time structure can yield good 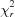 fits of the data.

While this is valuable for standard synchrotron beamlines including SAXS/WAXS, XAS and XFM applications, it can also find application in traditionally mature fields such as protein crystallography and powder diffraction. This is especially important as the temporal collection of diffraction spots can combine with many orders of flux and brightness difference for central spots, the weakest reflections collected, and even the profile tails of the weak reflections; and linearity across these dynamic ranges is crucial to structural interpretation. The advances in detector technology and insight can also be dramatic in application to fundamental experiments such as tests of quantum electrodynamics using EBIT where a factor of two reduction in statistical uncertainty or an improvement in linearity can probe new details of the universe (Chantler *et al.*, 2000 ▶); and in heavy ion storage rings where temporal structure is also often complex and matching this with the detector chain could be invaluable (Chantler *et al.*, 2007 ▶). Of course, recent popular developments with UV and X-ray free-electron lasers have complex and interesting temporal structure as well, arguing for the need for optimal matching of detector chains (Epp *et al.*, 2010 ▶). Importantly, there have been recent proposals to join some of these complex sources to investigate fundamental and applied problems in a coordinated manner, for example by merging a synchrotron beamline with an electron-beam ion trap (Chantler *et al.*, 2009*b* ▶; Simon *et al.*, 2009 ▶; Hutton *et al.*, 2009 ▶).

The resultant spectra will include complexities from the pulse of the fill pattern of the ring, from the usual monochromator optics, but especially from the unique characteristics of the EBIT geometry and source, and even more specifically from the opportunities for temporal pump–probe geometries. The development of these current ideas and their implementation in routine and *avant garde* experimental configurations will be an important objective.

Future detector fabrication featuring pixel dimensions of 75 µm square (Dinapoli *et al.*, 2010 ▶) will afford a factor of five reduction in flux per pixel for a given flux per mm^2^. The smaller pixel size will naturally improve resolution for many imaging applications. If the linearity and maximum count rate limits are similarly scaled, this will be a great opportunity for high linearity in large flux ranges.

However, poor agreement between experimental data and theoretical models is evident, in the sense of reliable 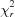 over high-flux regimes and especially in the region where dead-time dominates and the function ceases to be monotonic. Much improved fits are achieved if bunched spacing and shaping times are free parameters within some models, for a range of conditions. Others are not reasonably fitted by any model. This indicates at least one incorrect assumption in (all) the modelling approaches. The model dependence is complex, and the discriminant between model assumptions is sometimes weak, especially where the dominant literature models are unsuccessful, and despite useful empirical fits which by eye appear sound. Model validation post turn-over revealed a rate-dependent divergence. We found this an exciting opportunity to understand advanced detector linearity for the first time, for which this investigation was a major step forward. We suspect that there will be multiple causes of the current discrepancies including the difference between an idealized detector response and that of a realistic and complex detector system. A simple suggestion is to investigate Monte Carlo methods. We have (Trueb *et al.*, 2012 ▶), and the corresponding implementations, while correctly implemented, provide no additional insight nor success in this area.

Future work will involve model development to more fully account for experimental results, especially including single photon and Poissonian clustering with temporal fill patterns or bunch cycling times.

A better understanding and control of this matching of temporal structure and detector processing will yield:

(i) Optimized detector linearity (relative accuracy over a discrete range).

(ii) Maximal count rate in high-flux systems (optimized peak value).

(iii) A larger range of usable incoming photon rates.

(iv) Higher efficiency and lower statistical uncertainty in many applications.

## Figures and Tables

**Figure 8:**
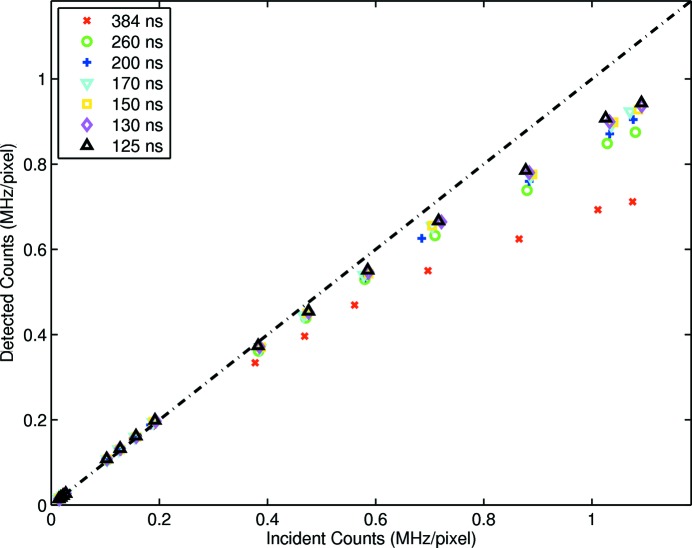
Enlargement of Fig. 4 ▶ to reveal the large linear range of the detector. Linearity to within approximately 2% is evident at short shaping times up to approximately 0.59 MHz pixel^−1^.

**Figure 9:**
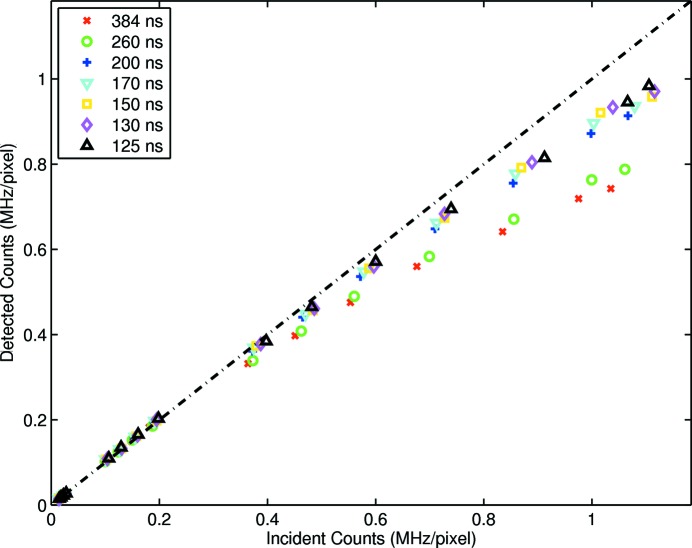
Enlargement of Fig. 5 ▶ to reveal the large linear range of the detector after optimization. Linearity to within approximately 2% is evident at short shaping times up to approximately 0.71 MHz pixel^−1^.

**Table 1:** Key parameters of the Australian Synchrotron storage ring

Energy (GeV)	3.0
Circumference (m)	216
Harmonic number	360
Revolution time (ns)	720.5
Revolution frequency (MHz)	1.388
Nominal current (mA)	200

**Table 2:** Fill parameters for the patterns shown in Fig. 1 ▶

	User fill	180 ns bunched	240 ns bunched
Rise time	140 ± 10 ns	2 ± 0.5 ns	2 ± 0.5 ns
Peak	0.9 ± 0.1 ns	1 ± 0.1 ns	1 ± 0.1 ns
Fall time	140 ± 10 ns	3 ± 0.6 ns	3 ± 0.6 ns
Period	720 ± 1 ns	180 ± 1 ns	240 ± 1 ns

**Table 3:** The seven nominal shaping times investigated

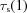	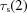	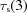	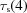	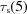	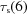	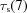
384 ns	260 ns	200 ns	170 ns	150 ns	130 ns	125 ns
